# Combination of specific single chain antibody variable fragment and siRNA has a synergistic inhibitory effect on the propagation of avian influenza virus H5N1 in chicken cells

**DOI:** 10.1186/s12985-014-0208-x

**Published:** 2014-11-29

**Authors:** Shuang Wang, Peng Zhang, Fei He, Ji-Gui Wang, Jia-Zeng Sun, Zhi-Li Li, Bao Yi, Ji Xi, Ya-Ping Mao, Qiang Hou, Dao-Li Yuan, Zi-Ding Zhang, Wei-Quan Liu

**Affiliations:** State Key Laboratory of Agrobiotechnology, Department of Biochemistry and Molecular Biology, College of Biological Sciences, China Agricultural University, Beijing, 100193 China; State Key Laboratory of Brain and Cognitive Sciences, Institute of Biophysics, Chinese Academy of Sciences, Beijing, 100101 China

**Keywords:** AIV H5N1, scFv, siRNA, Synergistic inhibitory effect

## Abstract

**Background:**

The avian influenza virus (AIV) causes frequent disease with high morbidity and mortality. RNA interference (RNAi) has been shown to provide an effective antiviral defense in animals, and several studies have focused on harnessing small interfering RNAs (siRNAs) to inhibit viral infections. In addition, single chain variable fragments (scFvs) contain the complete antigen binding site, and specific scFvs can bind to and neutralize viruses.

**Results:**

Fourteen positive scFvs were selected by the yeast two-hybrid system. Using molecular docking technology, we selected the three highest affinity scFvs for further functional validation. Results of indirect ELISA and IFA showed that all three scFvs could bind to FJ13 strain and had neutralizing activity, decreasing the viral infectivity markedly. Chicken fibroblastic DF-1 cells were transfected with scFvs in combination with siRNA-NP604 (an siRNA of anti-AIV NP protein previously reported). Following infection with FJ13 virus, copy numbers of the virus were significantly reduced from 12 h to at least 60 h post-infection compared to that achieved in cells transfected with scFv or siRNA-NP604 separately.

**Conclusions:**

A novel combination of antiviral siRNAs expressed in chicken cells and chicken antibody single-chain variable fragments (scFvs) secreted from the cells has a synergistic inhibitory effect on the avian influenza viral proliferation *in vitro*. Intracellular application of scFvs and anti-viral siRNA may provide a new approach to influenza prevention and treatment.

## Introduction

Avian influenza viruses (AIVs) are important pathogens affecting the poultry industry worldwide, with some infecting humans with a high fatality rate [1]. Due to the diversity and propensity for inter-species transmission, AIVs present a challenge for vaccine developers [2,3]. RNA interference (RNAi) has been shown to provide an effective antiviral defense in animals, and several studies have focused on harnessing small interfering RNAs (siRNAs) to inhibit viral infections [4–8]. An siRNA-based microbicide has been shown to protect mice from lethal herpes simplex virus 2 infection or transmission [9], and siRNAs specific for conserved regions of influenza virus genes prevented and even treated influenza viral infection in mice [6]. In 2011, Lyall et al. [3] reported that transgenic expression of an RNA hairpin molecule was capable of inhibiting influenza viral polymerase activity in the chicken.

ScFvs, another effective countermeasure against animal virus, are among the most widely used recombinant antibodies (rAbs) as they have been successfully modified into a number of different Ab formats and are easily expressed by several expression systems. Since they contain the complete antigen binding site, specific scFvs can bind to and neutralize viruses [10–12]. Ascione et al. [13] used a biopanning based approach to isolate a large array of scFv clones against H5N1 virus from the human semi-synthetic ETH-2 phage antibody library of which two, AV.D1 and AV.C4, exerted a significant inhibition of H5N1 virus infection. Additionally, a high-affinity human scFv antibody against the recombinant H5N1 virus hemagglutinin ectodomain (HA1) showed satisfactory antiviral effects against challenge with H5N1 viruses in embryonated chicken eggs [14].

In the present study, we report that use of siRNA targeting the NP protein, in combination with single chain variable fragments (scFvs) of anti-HA protein antibody, has a synergistic inhibitory effect on the propagation of avian influenza virus H5N1. Two antiviral genes were expressed in the DF-1 chicken fibroblast line such that an siRNA was stably expressed in the cells in combination with scFvs that were continuously secreted into the extracellular fluid. We show that the infectious titer and copy number of virus particles were both significantly reduced in infected cells.

## Results

### Selection of scFvs using the yeast two-hybrid (Y2H) method

**Figure 1:**
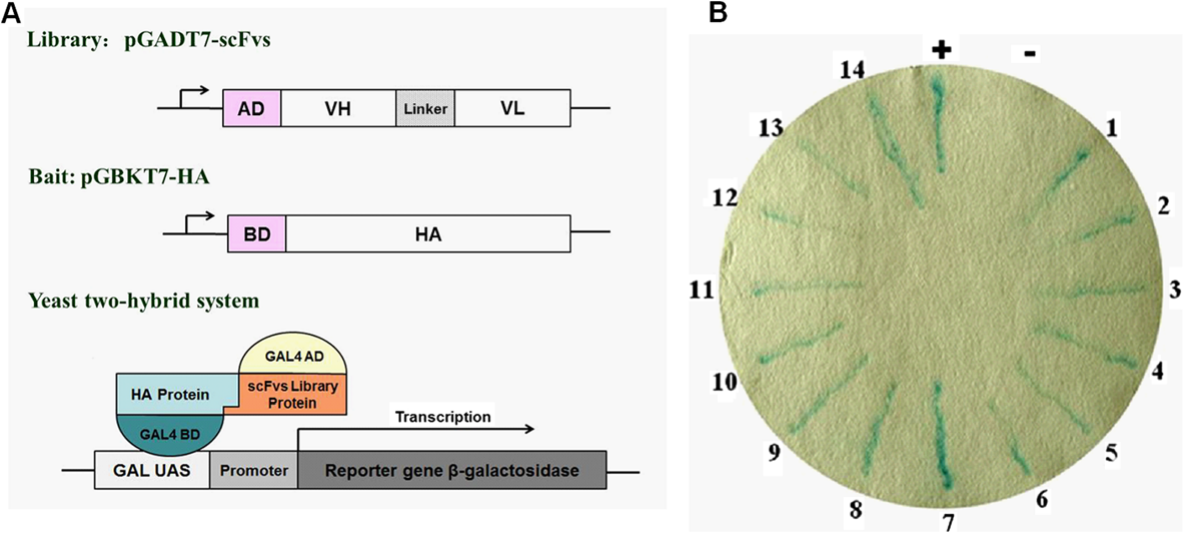
**Yeast two-hybrid assay and chromogenic results. (A)** Constructs used in the yeast two-hybrid assays for screening of specific scFvs against HA protein. A cDNA library of scFvs was subcloned into a pGADT7 yeast two-hybrid prey vector for fusion to the GAL4 transactivation domain (AD). **(B)** Screening for specific scFvs against HA. Yeast clones containing bait and prey vectors were selected on SD/-Leu/-Trp/-His/-Ade plate. Fourteen positive scFv fragments appeared blue through X-gal staining.

Initially, high yields of chicken immunoglobulin VH and VL gene fragments were obtained by PCR amplification. The joining of VH and VL via a flexible peptide (Gly_4_Ser)_3_ linker sequence using overlap extension PCR produced a pGADT7 scFv library. The library titer was 6.53×10^9^ cfu/ml. Before the Y2H experiments, it was first confirmed that pGBKT7-HA+pGADT7-BD and pGADT7-ScFv+ pGBKT7-blank had no autoactivator activity in yeast, and that growth of yeast cells did not impact the expression of pGBKT7-HA. Following incubation for 20 h to permit mating of pGBKT7-HA and pGADT7-ScFv in YDPA/Kana^+^, the appearance of a cloverleaf structure formed by yeast clustering confirmed that the mating was successful. Fourteen colonies positive for scFvs were identified by their blue color resulting from X-gal staining of β-galactosidase activity (Figure 1).

### Further screening of scFvs by computational prediction

**Figure 2:**
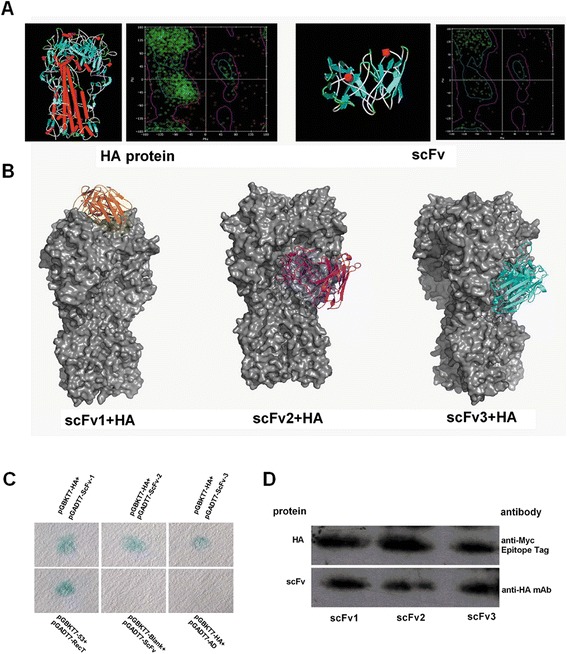
**Screening of scFvs by ZDOCK module of DiscoveryStudio software. (A)** Homology modeling of HA protein and scFvs. **(B)** Molecular docking between scFvs and HA protein. **(C)** The high-affinity scFvs were confirmed again by yeast two-hybrid assay. Chromogenic results showed scFv1, scFv2 and scFv3 were all positive and the blank controls were negative. **(D)** Expression of HA and scFvs in yeast as detected by western blot. Upper row: detection of HA expression by anti-Myc Epitope Tag.; Lower row: detection of scFvs expression by anti-HA Epitope Tag.

We utilized computational methods to predict the potential binding affinities between these antibodies and the HA protein. Three-dimensional structures of the HA protein and scFvs were constructed by DiscoveryStudio software. As can be seen in the Ramachandran plot, 95% of plots that were surrounded by the red trace contour indicate that the structures were highly reliable (Figure 2A). Binding affinities between the 13 scFvs and an HA protein were predicted by the ZDOCK module of the DiscoveryStudio package. As shown in Table 1, the docking results were graded and sorted in ascending order of binding energy. The 3 lowest binding energies were shown by Nos. 1, 2 and 6 which mean they had the highest affinities for the HA protein. These three scFvs (named scFv1-3) were chosen for the next functional tests. Docking results showed that they bound to separate conformational epitopes (Figure 2B). The Y2H results showed good reproducibility and all were expressed in the mating yeast (Figure 2C and D). The three sequences were all submitted to GenBank [ID: FJ605117, FJ605118, FJ605119].

**Table 1:** **Binding values of HA protien and positive scFvs**

**Top ordering**											**Binding energy**
1	-2.094395	0.294107	1.59388	3	178	135	19.82	2	-1	-1	-154.472
2	-0.733038	1.524811	-2.844718	8	29	206	21.7	26	6	-1	-149.158
6	-0.733038	0.997766	-2.693871	163	3	183	22.6	6	73	1	-137.171
5	-0.314159	0.632797	1.989858	201	185	47	20	2	-1	-1	-131.664
11	-1.570796	1.554776	0.100351	10	23	2	22.56	1	-1	-1	-118.933
3	-1.989675	0.934873	-2.010822	162	187	2	20.52	12	21	-1	-116.42
13	-0.523599	1.135813	-2.658866	178	9	174	19.24	1	-1	-1	-116.378
4	-0.733038	1.743328	0.663347	19	164	189	19.84	2	-1	-1	-116.115
7	-1.675516	0.65244	2.197129	8	33	178	19.38	3	-1	-1	-114.846
8	-0.837758	1.889815	2.70157	14	22	21	24.82	1	-1	-1	-111.05
14	1.832596	0.41964	2.974122	14	3	135	22	1	-1	-1	-110.35
9	-0.314159	0.869748	2.095969	183	159	16	23.3	2	20	1	-107.702
12	2.094395	1.736875	3.023612	4	39	183	19.78	1	-1	-1	-107.003
10	2.879793	1.184361	0.625864	19	21	0	18.7	1	17	-1	-82.9037

### Expression of the three scFvs in DF-1 cells

**Figure 3:**
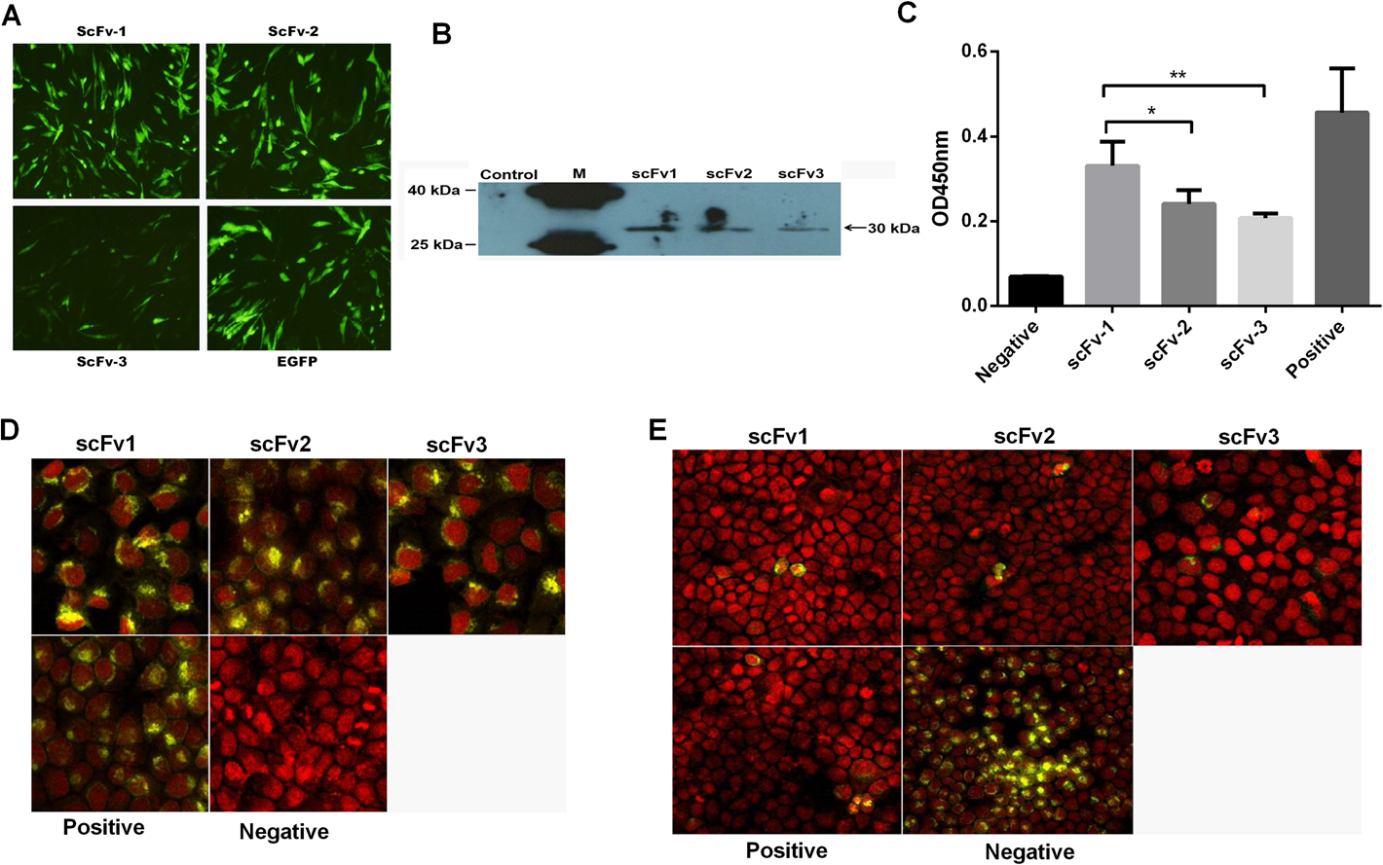
**Verification of the binding activity of scFvs expressed by DF-1 to FJ13 virus. (A)** Transient expression of scFvs in serum-free cultured DF-1 cells indirectly detected by EGFP. **(B)** Western blot assay used to directly detect the transient expression of scFvs (~30 KDa) in serum-free cultured DF-1 cells. **(C)** Indirect-ELISA assay used to assess the titer of scFvs binding to HA protein. **(D)** IFA used to detect the virus binding activity of scFvs. After infection by FJ13 virus (yellow) for 36 h, MDCK cells (red) were subjected to IFA using scFv1, scFv2, scFv3 and monoclonal antibody against AIV H5N1 subtype HA protein as primary antibodies. The positive control used the 1:300 anti-AIV H5N1 HA mAb and the negative used antibody diluent as the primary antibodies. **(E)** Virus neutralization of ScFvs, detected by IFA. The scFvs and anti-AIV H5N1 HA mAb (1:30, as the positive control) were incubated with FJ13 virus (200CCID_50_, yellow ) respectively for 1h before infection of MDCK cells (red). The negative control used antibody diluent instead. Anti-AIV H5N1 HA mAb was used as the primary antibody. In **(D)** and **(E)**, the cells were counterstained with PI (red). Data are from 3 independent experiments (mean ± SD). Statistical significance was analyzed by Student’s t test; *P <0.05; ** P <0.01; ns, not significant.

Signal peptides and c-Myc Epitope Tag sequences were respectively added to the 5′ and 3′ ends of the scFvs through PCR primers (Table 2), and the modified scFvs were inserted separately into pIRES2-EGFP vector. At 48 h following transfection of DF-1 cells with the pIRES2-scFvs, the expression level of GFP was readily observed by fluorescence microscopy (Figure 3A). Following the culture supernatants in each well were concentrated and western blotting showed that all three scFvs were expressed (Figure 3B).

**Table 2:** **Sequences of oligonucleotides used for construction of libraries**

**Primer**	**Nucleotide sequence (5 to 3′)**
RT-VH	GGAGGAGACGATGACTTC
RT-VL	TTATAGGACGGTCAGGGTTGTC
VH-P1	ATGAGCCCACTCGTCTC
VH-P2	AGAGCCACCTCCGCCTGAACCGCCTCCACCGGAGGAGACGATGACTTC
VL-P1	GGCGGAGGTGGCTCTGGCGGTGGCGGGTCGGCGCTGACTCAGCCGT
VL-P2	TTATAGGACGGTCAGGGTTGTC
VHp1-NdeI	GGGAATTCCATATGGCCGTGACGTTGGAC
VLp2-BamHI	CGCGGATCCTTATAGGACGGTCAGGGTTGTC
IRES-P1	**AGATCT** *ATGAGCCCACTCGTCTCCTCCCTCCTGCTCCTGGCCGCCCTGCCAGGGCTGATGGCG*
	GCCGTGACGTTGGACG
IRES-P2	TTA*CAGATCCTCTTCAGAGATGAGTTTCTGCTC* **GCCGGC**TAGGACGGTCAGGGTTG

Synthetic linker used for overlap extension PCR shown underlined.

Restriction enzyme sites used for cloning shown in bold.

Signal peptide sequence and c-Myc epitope tag sequence shown italics.

### The binding activity of scFvs to FJ13 virus

The binding activity of scFvs to virus in cells was assessed using indirect-ELISA and IFA. As tested by ELISA, all 3 scFvs expressed by DF-1 bound to the FJ13. The titer of scFv1 was slightly higher than the others, but the difference was not statistically significant (Figure 3C). The IFA used the three scFvs as the primary antibodies. Results showed that all three exhibited virus binding activity in MDCK cells. Titers of the scFvs at 1 μg/ml were equal to the anti-AIV H5N1 HA mAb at 1:300 (Figure 3D). Mixing virus with the scFvs 1 h before infection of cells at 37°C significantly reduced its infectivity (Figure 3E), indicating that all three scFvs had virus neutralizing activity.

### Effect of siRNA in combination with scFv1 against FJ13 virus in DF-1 cells

**Figure 4:**
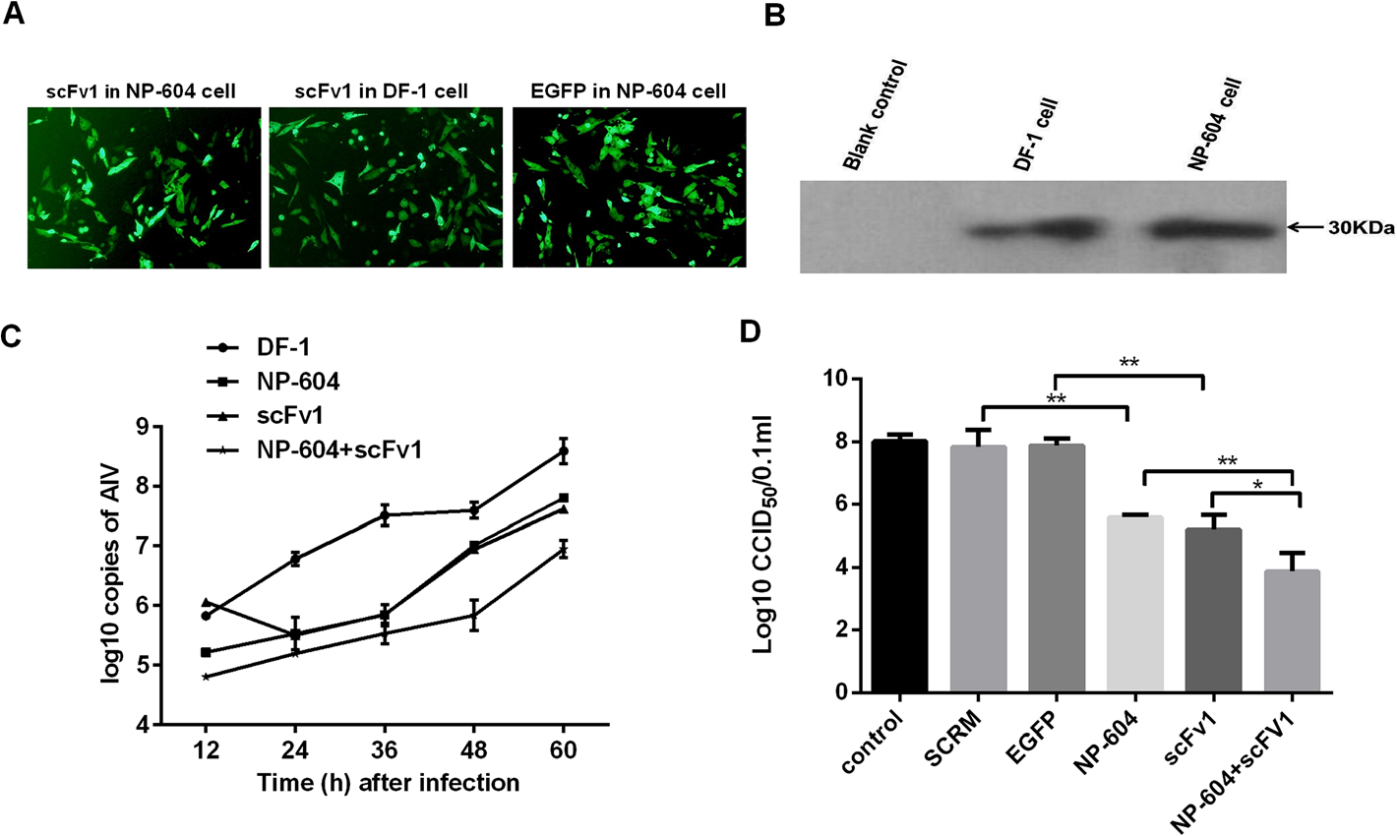
**siRNA in combination with scFv1 against FJ13 virus in DF-1 cells. (A)** Transient transfection of NP604 and DF-1 cells by pIRES2-scFv1. The negative control was transfected by pIRES2-EGFP. **(B)** Expression of scFv1 in NP604 and DF-1 cells detected by western blot. **(C)** mRNA levels of AIV FJ13 analyzed by qPCR from 24 h to 60 h p.i. in DF-1, NP604, scFv and scFv-NP604 cells. All measurements were normalized to expression of β-actin genes, and the copies of HA gene mRNAs were quantified by comparison with a standard curve derived from known amounts of pEASY-HA. **(D)** All cells were infected with 100 CCID_50_ of FJ13. Virus titers of supernatant samples collected at 60 h p.i. were determined as CCID_50_ in normal MDCK cells. Data are from 3 independent experiments (mean ± SD). Statistical significance was analyzed by Student’s t test; *P <0.05; **P <0.01; ns, not significant.

From the above results, scFv1 was selected to be co-expressed with siRNA NP604 targeting the NP of FJ13 [15], and DF-1 cells were stably transfected with the shRNA-expression plasmid psiSTRIKE-NP604 (hereafter referred to as NP604 cells). Expression of GFP in the NP604 cells was observed by fluorescent microscopy 48 h following transient transfection with pIRES2-scFv1 (Figure 4A). The cell culture supernatant was concentrated and western blots showed that the scFv1 was also expressed in the NP604 cells (Figure 4B).

Infection of DF-1 and MDCK cells with FJ13 virus confirmed cytopathogenicity in DF-1 cells, with similar virus titers (as cell culture ID_50_ assay, CCID_50_). The difference in FJ13 virus growth kinetics in each cell line was evaluated by qPCR. First, DF-1 and NP604 cells were transiently transfected with pIRES2-ScFv1 (hereafter referred to as scFv cells and scFv-NP604 cells respectively). Copy numbers of the viral genomic RNA were then determined by qPCR every 12 h for 60 h in DF-1, NP604, scFv and scFv-NP604 cells infected with FJ13 virus. As shown in Figure 4C, although the growth of FJ13 in NP604 and scFv cells was significantly lower than in DF-1 cells, it was consistently slightly lower in the scFv-NP604 cells than in all others. The results by CCID_50_ assay were similar to qPCR (Figure 4D).

## Discussion

ScFvs consist of VH and VL chains joined via a flexible peptide linker. Their advantages are high antigen binding activity, expression efficiency, strong penetrability and a short *in vivo* half-life [11]. The scFvs producted by murine hybridoma cell lines are capable of binding target antigens with an affinity similar to that of the parent mAb [16]. In addition, the single VH and VL region of the chicken immunoglobulin gene simplifies building a library, in contrast to that of mammals [17,18]. We generated VH and VL fragments from AIV H5N1 FJ13 immunized chickens. Assembly of VH, VL and linker fragments by overlap extension PCR yielded a library with a titer of 6.53×10^9^ cfu/ml, high enough for the Y2H screen.

For generating synthetic scFvs, several different molecular display formats have been described, including phage-display, ribosome display and cell-surface display [11]. The yeast two-hybrid system is a simple, cost-effective technique to screen for the scFv library, and antibodies synthesized in this system generally exhibit good expression levels and specific binding activities in eukaryotic cells [19]. In a comparative study using the same immune scFv cDNA library, yeast display was shown to sample the immune antibody repertoire considerably more fully than phage display, selecting all the scFv identified by phage display and twice as many novel antibodies [20]. However, Y2H library screening also generates a significant number of false-positive interactions, including “biological” false-positives: protein–protein interactions that occur in yeast cells, but do not occur *in vivo* in the organism of study, and “technical” false-positives: protein–protein interactions identified in Y2H screens due to technical limitations of the system [21]. It is very important, therefore, that a verification test be conducted to confirm the specificity of scFvs binding to the HA protein. The number of positive clones screened by Y2H is too large and difficult to verify individually, and using bioinformatics software for further screening greatly reduced the labor intensity. By ZDOCK software analysis, we chose the three with lowest HA binding energy from the 14 Y2H positive ones. The results of functional verification indicated Y2H combined with ZDOCK software analysis was reliable and effective.

To detect the co-antiviral effect induced by siRNA in combination with scFv1, NP-604 cells were transiently transfected with the scFv1 expression plasmid. Virus titers in the culture supernatants of NP604 cells transfected with scFv1 were reduced by 70-fold compared with titers in NP604 cells or scFv1 cells alone at 60 h post inoculation. Our study opens a new approach to influenza prevention and treatment by using scFv and siRNA together. We predict this concept can also provide a basis for breeding transgenic AIV-resistant chickens. As transgenic siRNAs would be stably expressed in all body cells, they will be present to interfere with NP synthesis and limit viral replication upon viral invasion. Since scFvs are secreted into the blood after expression, they will bind to the HA of circulating virus, thereby preventing viral infection and spread. If they are efficiently expressed, the result might be significant inhibition of virus at the earliest stage of infection, before an acquired immune response can be mounted.

## Conclusions

Three scFvs binding to the HA protein of AIV FJ13 strain with high affinity were selected by the yeast two-hybrid system and molecular docking technology. A combination of antiviral siRNAs expressed in chicken cells and chicken antibody single-chain variable fragments (scFvs) secreted from the cells has a synergistic inhibitory effect on virus proliferation *in vitro*. Intracellular expression of scFvs and siRNA against virus suggests a new approach to influenza prevention and treatment.

## Methods

### Virus and cells

Influenza A virus strain A/duck/Fujian/13/2002 (FJ13), H5N1 subtype, was kindly provided by the late Professor Hongwei Gao (Academy of Military Medical Sciences, Changchun). The AIV was propagated in the allantoic cavity of 10-day-old embryonated specific pathogen-free (SPF) eggs. MDCK and DF-1 cells were cultured in Dulbecco’s modified Eagle’s medium (DMEM, Gibco) supplemented with 10% fetal bovine serum (FBS, Gibco) at 37°C in 5% CO_2_ [22]. All experiments with H5N1 virus were conducted in a biosafety level 3 laboratory facility at the Changchun Institute of Veterinary Science.

Virus titration (cell culture ID_50_ assay, CCID_50_) was carried out in 96-well microtitration plates (Costar) seeded with MDCK cells (10^5^ cells per well). Serial 10-fold virus dilutions in DMEM (100 μl) were made in triplicate and the plates were incubated in 5% CO_2_ at 37°C. Wells were examined microscopically and endpoints were taken as the inverse of the highest dilution showing CPE. Titers were calculated by the Reed-Muench method.

### Construction of scFv Libraries

Eight four-week-old SPF chickens were immunized by four injections of formalin-inactivated FJ13 virus four times, together with complete Freund’s adjuvant for the 1^st^ injection and incomplete adjuvant thereafter. The time interval until the second injection was two weeks, and one week for the others. Virus protein concentrations of the respective injections were 0.2 mg, 0.4 mg, 0.8 mg and 0.8 mg. Seven days later, the chickens were euthanized and spleens were removed for total RNA extraction. Antibody titers in serum from the wing vein were determined by the hemagglutination-inhibition test.

Total RNA was extracted from homogenized spleens with Trizol (Invitrogen, USA) (Figure 1A). Approximate 8 μg RNA was randomly primed in a total volume of 100 μl to produce complementary DNA (cDNA) using a cDNA synthesis kit (Trans, China). The specific primers were RT-VH or RT-VL (Table 2).

VH and VL chains were amplified using primers VH-P1 &VH-P2 for the VH region or VL-P1 & VL-P2 for the VL region (Table 2). Gel-purified VH and VL chains were connected via a synthetic linker (Table 2) using overlap extension PCR. After 16 rounds of amplification (94°C for 1 min, 45°C for 1 min and 72°C for 1 min), 2 μl of each primer VHp1-NdeIand VLp2-BamHI (Table 2) were added and an additional 14 cycles was performed. The scFv genes were ligated with the pGADT7 vector ((kindly provided by Xing-Feng Liu Ph.D. of Tsinghua University).

### Yeast two-hybrid method for scFvs selection

The HA protein of FJ13 was used as bait protein to test the pGADT7-scFv library in a yeast two-hybrid (Y2H) screen. Plasmid pGBKT7-HA was prepared by cloning RT- PCR fragments of the FJ13 virus genome RNA into pGBKT7 vector. The AH109 yeast strain was co-transfected with the bait construct and empty pGADT7 vector to test and exclude auto-activation of reporter genes. After verification, the Y187 yeast strain was transfected with pGBKT7-HA. The Y2H screen was performed on HA and scFvs gene libraries using pGBKT7-HA and pGADT7-ScFv (Figure 1A). Briefly, the pre-transformed scFvs library in the AH109 yeast strain was mixed and mated with Y187 containing pGBKT7-HA. After 24 h, the culture was spread on SD/-Leu/-Trp plates and the surviving colonies were further verified on SD/-Leu/-Trp/-His/-Ade medium. After 3 days, the colonies were streak cultured on a new SD/-Leu/-Trp/-His/-Ade plate for 2 days. A sterile filter paper was fitted closely over the plate which had previously been dipped into Zbuffer/X-gal (Sino-American, China). The positive clones gradually turned blue between 30 min and 8 h.

### Homology modeling and molecular docking

Both computational predictions were performed with DiscoveryStudio package with default settings. The template for HA protein was 2IBX (sequence identity = 96%) [23]. While the template for the antibodies was 1AQK_H (sequence identity = 53%) [24]. The MODELLER module [25] was used for homology modeling while the ZDOCK module was used for the prediction of interactions between HA protein and antibodies [26].

### Cells transfection

Chicken DF-1 cells were stably transfected with the shRNA_expression plasmid psiSTRIKE-NP604 (Target sequence: AATGATCGGAATTTCTGGAGA) [15], using Lipofectamine™ 2000 reagent (Invitrogen). The transfected cells were selected with 800 μg/ml G418 (Merck) for 3 weeks with medium changed every 48 h. Individual colonies were picked and amplified in the same medium with 400 μg/ml G418. Transfectants expressing the shRNA (NP604 cells) were maintained as cell lines.

ScFv1, scFv2 and scFv3 were cloned into the BglII and EcoRI sites of bicistronic expression vector pIRES2-EGFP (Clontech) to generate pIRES2-scFv1, pIRES2-scFv2 and pIRES2-scFv3 respectively. DF-1 and NP604 cells were transiently transfected with the pIRES2-scFvs using Lipofectamine™ 2000 according to the manufacturer’s instructions.

### Western blotting

Following transfection, culture supernatants containing scFvs were collected and concentrated in Millipore filter tubes (Amicon-Ultra-15 MWCO 10KD). The primary antibody was mouse anti-c-Myc Epitope Tag mAb (MBL: 1:1000 dilution) and the method details have been given previously [27].

### Indirect-ELISA

FJ13 virus purified by polyethylene glycol (PEG) precipitation was adjusted to a concentration of 5 μg/ml in phosphate buffered saline (PBS, pH7). A total of 100 μl per well was used for binding to 96 well plates overnight at 4°C. The three scFvs diluted to 1 μg/ml with antibody diluent (PBST/ 0.1% BSA) were added to separate wells as primary antibodies. Positive control wells consisted of 100 μl Anti-AIV H5N1 HA mAb (1:300; a gift from Professor Sun Ming) as well as antibody diluent alone as a negative control. Mouse anti-c-Myc Epitope tag mAb (MBL) diluted 1:1000 was the secondary antibody, and goat-anti-mouse IgG HRP (1:2000) (MBL) the third. The method details have been given previously [15].

### Indirect immunofluorescence assay

MDCK cells were washed 3 times with PBS and fixed in cold fixative solution (acetone: methanol, 3:1) for 10 min at 4°C followed by 3 washes with PBS and incubation for 1 h at 37°C with individual primary antibodies. The cells were washed 3 times and incubated with purified primary mouse anti-c-Myc Epitope Tag mAb (MBL: 1:1000 dilution) as the second antibody. The cells were again washed 3 times, incubated with fluorescein isothiocyanate (FITC)-conjugated goat anti-mouse IgG antibody (Sigma, 1:100 dilution) for 1 h at 37°C, washed a further 3 times with PBS, and stained with 100 μg/ml propidium iodide (PI) for 5 min. Fluorescence was observed with a confocal laser scanning microscope (Nikon TE2000-E).

### Real-time quantitative PCR (qPCR) analysis

For quantitation of AIV replication in different cells from 12 h to 60 h (sampling every 12 h) following infection with 100 CCID_50_ FJ13, total RNA was extracted from 250 μl cell lysate supernatants using Trizol reagent (Invitrogen), and qPCR reactions were performed using Faststart SYBR Green mixture (with ROX; CWBIO) and the Roche 480 real-time PCR detection system. Thermal cycle conditions were initial denaturation at 94°C for 3 min, followed by 40 cycles at 94°C for 10 s, 60°C for 30 s, and melting curve at 95°C for 15 s and 60°C for 15 s. The specific primers used for identification of the HA gene were: 5′-GCCATTCCACAACATACACCCTC-3′ (sense) and 5′-TTCCCTGCCATCCTCCCTC-3′ (antisense). Cellular β-actin gene was quantified as an internal control. The copies of HA mRNAs were quantified by comparison with a standard curve derived from known amounts of pEASY-HA plasmid (Trans, China).

## Highlights

1. We reported a novel combination of anti-AIV H5N1 siRNA and scFv.
2. Anti-AIV siRNA was expressed in the cells and scFv was secreted from the cells.
3. This combination has a synergistic inhibitory effect on virus proliferation.
4. Using bioinformatics software and Y2H for screening scFvs reduced labor intensity.
